# Supplementary material for: Drug repurposing for aging research using model organisms
Source: Aging Cell. 2017 Jun 16;16(5):1006–15. doi: 10.1111/acel.12626 (PMC5595691; doi:10.1111/acel.12626)
Supplement: Supplementary file 7 — Data S1 Zip‐Archive of all report cards. [file ACEL-16-1006-s007.zip › RC_STR.pdf]

STR

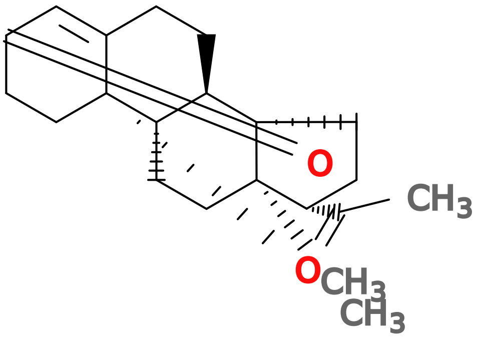

Database identifiers

ChEMBLCompound	CHEMBL103
DrugBank	DB00396
CHEBI	17026
ZINC	ZINC04428529
eMolecules	29549914
eMolecules	511682

Ranking

	Rank	Score
Drosophila	133/697	0.762
C. elegans	NA	NA

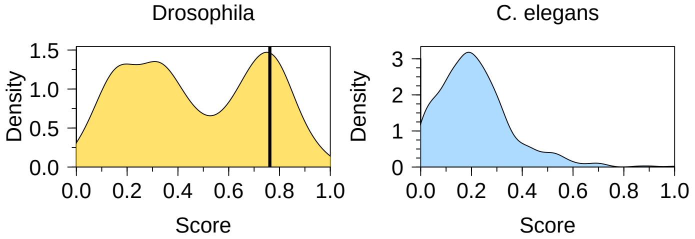

	Ageing implication	Domain conservation	Binding site conservation	Binding affinity	Bioavailability	Lipinski	Promiscuity	Purchasability	Drug approval	**Total**
Drosophila	1.0	0.84	0.88	0.845	(0.9)	0.0	-0.0	0.1	0.1	0.762
C. elegans	NA	NA	NA	NA	NA	NA	NA	NA	NA	NA

Names

- **progesterone**
- (S)-Pregn-4-en-3,20-dione
- 4-Pregnene-3,20-dione
- Agolutin
- corpus luteum hormone
- Crinone
- Delta(4)-pregnene-3,20-dione
- Endometrin
- Gelbkoerperhormon
- Gesterol
- luteohormone
- Progestasert

· Progestin
· Prometrium
· Regumate
· Syngestrone

Roles

progestin, contraceptive drug, human metabolite, progesterone receptor agonist, Progesterone receptor agonist

Status

Approved drug (according to ChEMBL)	Yes
Administration Route	Oral, Intravaginal, Intramuscular
Number of Rule of 5 violations	0
Binding affinity to original target in log units (RF-Score prediction)	6.69
Burns *C. elegans* bioavailability prediction	-2.54

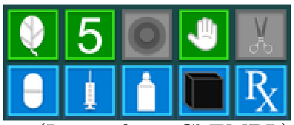

(Image from ChEMBL)

Medical Information

Indicatation: For progesterone supplementation or replacement as part of an Assisted Reproductive Technology (ART) treatment for infertile women with progesterone deficiency and for the treatment of secondary amenorrhea. Also used for the reduction of the incidence of endometrial hyperplasia and the attendant risk of endometrial carcinoma in postmenopausal women receiving estrogen replacement therapy, as well as treatment of abnormal uterine bleeding due to hormonal imbalance in the absence of organic pathology such as fibroids or uterine cancer.
Mechanism of action: Progesterone shares the pharmacological actions of the progestins. Progesterone binds to the progesterone and estrogen receptors. Target cells include the female reproductive tract, the mammary gland, the hypothalamus, and the pituitary. Once bound to the receptor, progestins like Progesterone will slow the frequency of release of gonadotropin releasing hormone (GnRH) from the hypothalamus and blunt the pre-ovulatory LH (luteinizing hormone) surge. In women who have adequate endogenous estrogen, progesterone transforms a proliferative endometrium into a secretory one. Progesterone is essential for the development of decidual tissue and is necessary to increase endometrial receptivity for implantation of an embryo. Once an embryo has been implanted, progesterone acts to maintain the pregnancy. Progesterone also stimulates the growth of mammary alveolar tissue and relaxes uterine smooth muscle. It has little estrogenic and androgenic activity.
Metabolism: Progesterone is metabolized primarily by the liver largely to pregnanediols and pregnanolones.
Retail: Abraxis BioScience Inc., APP Pharmaceuticals, Ascend Therapeutics, Carlisle Laboratories Inc., Catalent Pharma Solutions, Columbia Labs and 21 more suppliers
Prometrium 100 mg capsule - 1.69 USD / capsule
First-progesterone vgs 25 suppository - 2.88 USD / each
First-progesterone vgs 50 suppository - 2.93 USD / each
First-progesterone vgs 100 suppository - 3.0 USD / each
First-progesterone vgs 200 suppository - 3.15 USD / each
First-progesterone vgs 400 susuppositoryp - 3.33 USD / each
and 12 more options

(Information from DrugBank)

Compound Target Characteristics

Apolipoprotein D

Best gene implication in ageing for this target family came from gene Q9NAZ4 annotated in UniProt release 2014_02. Annotation GO 8340 (determination of adult lifespan) was Inferred from Mutant Phenotype

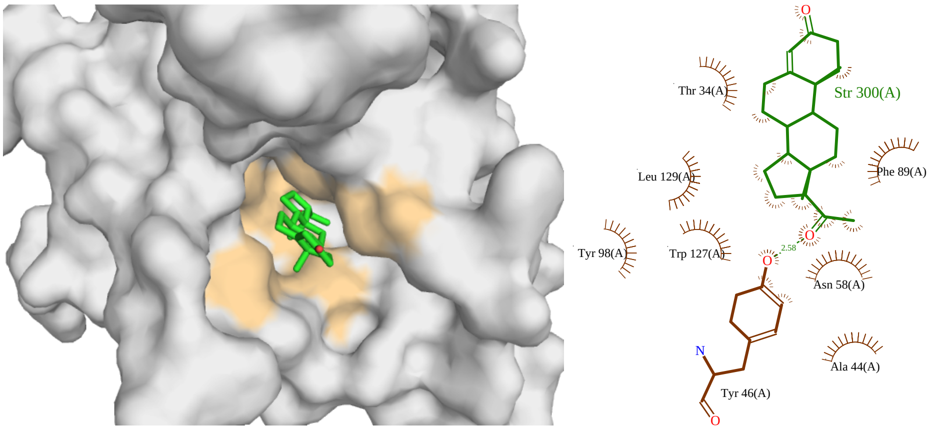

protein	amino acids contacts (binding site)
PDB:2hzq:chainA:P05090	T A Y N F Y W L
tr:C9JX71:C9JX71_HUMAN	T A Y N F Y W L
sp:P05090:APOD_HUMAN	T A Y N F Y W L
tr:C9JF17:C9JF17_HUMAN	T A Y N F Y W L
tr:M0R4S2:M0R4S2_RAT	V A Y N F Y W L
sp:P51910:APOD_MOUSE	A A Y N F Y W L
tr:Q9NAZ4:Q9NAZ4_DROME	F A Y N F Y W L
tr:Q8SXR1:Q8SXR1_DROME	F A Y N F Y W L

	whole protein		domain-based		contact-based	
protein	ident	simil	ident	simil	ident	simil
PDB:2hzq:chainA:P05090	0.96	0.98	0.96	0.98	1.0	1.0
tr:C9JX71:C9JX71_HUMAN	0.79	0.79	0.77	0.77	1.0	1.0
sp:P05090:APOD_HUMAN	1.0	1.0	1.0	1.0	1.0	1.0
tr:C9JF17:C9JF17_HUMAN	0.86	0.86	0.99	0.99	1.0	1.0
tr:M0R4S2:M0R4S2_RAT	0.72	0.91	0.74	0.91	0.88	0.92
sp:P51910:APOD_MOUSE	0.72	0.91	0.74	0.91	0.88	0.93
tr:Q9NAZ4:Q9NAZ4_DROME	0.32	0.65	0.38	0.77	0.88	0.88
tr:Q8SXR1:Q8SXR1_DROME	0.32	0.65	0.38	0.77	0.88	0.88

NLaz (FBgn0053126) associated phenotypes

chemical resistant, chemical sensitive, courtship behavior defective, long lived, oxidative stress response defective, progressive, short lived, starvation stress response defective, stress response defective

(Information from FlyBase)
